# Methylseleninic acid activates Keap1/Nrf2 pathway via up-regulating *miR-200a* in human oesophageal squamous cell carcinoma cells

**DOI:** 10.1042/BSR20150092

**Published:** 2015-10-08

**Authors:** Mei Liu, Chenfei Hu, Qing Xu, Lechuang Chen, Kai Ma, Ningzhi Xu, Hongxia Zhu

**Affiliations:** * Laboratory of Cell and Molecular Biology & State Key Laboratory of Molecular Oncology, Cancer Hospital, Chinese Academy of Medical Sciences & Peking Union Medical College, Beijing, PR China

**Keywords:** kelch-like ECH-associated protein 1 (Keap1), Krüpple-like factor 4 (KLF4), micro-ribonucleic acid-200a (miR-200a), methylseleninic acid (MSA), nuclear factor E2-related factor 2 (Nrf2), oesophageal squamous cell carcinoma (ESCC)

## Abstract

Oesophageal squamous cell carcinoma (ESCC) occurs at a very high rates in certain regions of China. There are increasing evidences demonstrating that selenium could act as a potential anti-oesophageal cancer agent, but the precise mechanisms involved are still not completely understood. Methylseleninic acid (MSA), as a potent second-generation selenium compound, is a promising chemopreventive agent. Previous studies demonstrated that the kelch-like ECH-associated protein 1 (Keap1)/nuclear factor E2-related factor 2 (Nrf2) system plays a critical role in cancer prevention, but little is known about its association with MSA in ESCC cells. In the present study, we observed that MSA treatment significantly down-regulated Keap1, induced nuclear accumulation of Nrf2 and enhance the antioxidant response element (ARE) promoter activity in ESCC cells. MSA could also significantly induce *miR-200a* expression and inhibit Keap1 directly. Antagomir-200a could attenuate MSA treatment-induced Keap1 down-regulation in ESCC cells. Moreover, MSA-induced *miR-200a* expression was dependent on the mediation of Krüpple-like factor 4 (KLF4). These results reaffirm the potential role of MSA as a chemopreventive agent via the regulation of KLF4/*miR-200a*/Keap1/Nrf2 axis in ESCC cells.

## Abstract (precis)

Methylseleninic acid (MSA), as a potent second-generation selenium compound, could activate KLF4/miR-200a/Keap1/Nrf2 pathway in oesophageal squamous cell carcinoma cells.

## INTRODUCTION

Selenium is an essential trace element with a lot of physiological functions in humans and animals. Many studies in animals and humans clinical trials have indicated that selenium is a promising chemopreventive agent for several major types of cancer, including oesophageal squamous cell carcinoma (ESCC) [1–4]. For instance, supplementation with selenium-containing compounds was associated with a significantly lower cancer mortality rate [4]. Selenomethionine had a protective effect against mild oesophageal squamous dysplasia at baseline [5]. However, the exact mechanism of selenium in the prevention of oesophageal carcinogenesis is still unclear.

Recent studies have shown that certain chemopreventive agents could active nuclear factor E2-related factor 2 (Nrf2) either by oxidative or covalent modification of its inhibitor kelch-like ECH-associated protein 1 (Keap1) or by phosphorylation of Nrf2 [6–8]. Nrf2, a redox sensitive transcription factor, is a master regulator of intracellular antioxidants and phase II detoxification enzymes by the transcriptional activation of many antioxidant response element (ARE)-containing genes [9,10]. Under homoeostatic conditions, Nrf2 is generally localized in the cytoplasm, where it is bound to Keap1. Once under oxidative stress or chemopreventive compounds, Keap1 acts as a molecular sensor and undergoes chemical modifications in a series of reactive cysteine residues, allowing the release of Nrf2, which escapes from degradation and translocates to the nucleus [11]. Previous study indicated that the Keap1/Nrf2 system regulates an important defensive mechanism against upper aerodigestive tract carcinogenesis, including cancers of the tongue and the oesophagus [12]. And, some selenium compounds have been shown to increase Nrf2 in prostate cancer [13]. The latest study reported that methylseleninic acid (MSA) treatment could up-regulate Nrf2 mRNA level in prostate tumour tissues [14]. However, the relationship between MSA and Nrf2 activation in ESCC has not been elucidated.

miRNAs as a type of endogenous non-coding RNA, mediate post-transcriptional gene regulation and participate in nearly all biological processes [15]. So far, a regulatory role for miRNAs in controlling Keap1 gene expression has been characterized in several studies [16–18]. Eades et al. [16] showed that *miR-200a* could target Keap1 directly and histone deacetylase (HDAC) inhibitor SAHA (suberanilohydroxamic acid, vorinostat) could induce re-expression of *miR-200a* in breast cancer cell lines. Furthermore, our previous study demonstrated that MSA could inhibit HDAC activity in ESCC cells [19]. All of these prompted us to hypothesize that MSA might regulate *miR-200a* to modulate Keap1/Nrf2 pathway in ESCC cells. To test this issue, we detected the expression level of *miR-200a* in ESCC cells with or without MSA treatment, respectively. We found that MSA treatment resulted in an increase in *miR-200a* expression and activation of Keap1/Nrf2 pathway. We also found that MSA treatment significantly increased Krüpple-like factor 4 (KLF4) binding to the *miR-200a* promoter region in ESCC cells.

## MATERIAL AND METHODS

### Cell culture, RNA isolation and quantitative real-time PCR

KYSE150, KYSE180, KYSE410 and KYSE510 were cultured in RPMI-1640 medium supplemented by 10% FBS at 5% CO_2_. Total RNA was isolated from cultured cells using TRIzol Reagent (Invitrogen) and reverse-transcribed to cDNA with M-MLV Reverse Transcriptase (Promega). Real-time PCR was performed on the StepOne Plus Real-Time PCR System (Applied Biosystems) with Power SYBR Green PCR Master Mix (Applied Biosystems), according to the manufacturer's protocol. The sequences of the PCR primers that were used to detect KLF4 and β-actin were reported previously [19].

### Plasmids, transfection and reagents

pGL3-ARE and pcDNA3-HA-Nrf2 were generous gifts from Professor Xiaoming Yang. The 3′-UTR of Keap1 was amplified using the following primers: 5′-TCATACTAGTGGCACTTTTGTTTCTTGGGC-3′ and 5′-GCATTAAGCTTCAGGGTGAAAGACACTAG-3′ and cloned into pMiR-Report vector (Ambion) digested with HindIII and Spe I. We also generated three bases mutation in the predicated target site for *miR-200a* by using a QuickChange site-specific mutagenesis kit (Stratagene). All constructs were sequenced in Sangon Company. Transfection of plasmids was performed in 70%–80% confluent cells using Lipofectamine 2000 Reagent (Invitrogen) according to manufacturer's protocol.

MSA was purchased from Sigma–Aldrich (Sigma–Aldrich Inc.). Pre-miR *miR-200a* precursor and Pre-miR negative control were purchased from Ambion. Antagomir-200a was synthesized from Ribobio. KLF4 siRNA and scramble control were purchased from OriGene (OriGene Technologies). Transfections of Pre-miR *miR-200a* precursor, Pre-miR negative control, KLF4 siRNA and scramble control were performed by using siPORT *NeoFX* Transfection Agent (Life Technologies) according to manufacturer's protocol.

### miRNA-specific quantitative real-time RT-PCR

For miRNA analysis from cultured cells, miRNA was isolated using a mirVana RNA isolation kit (Ambion). Reverse transcription and real-time PCR were performed as described [20] by using miRNA-specific quantitative real-time PCR (Applied Biosystems). The small RNA U6 was used as an internal control for normalization. Real-time PCR was performed using a StepOne Plus Detection System and fold changes in gene expression were calculated using the 2^−^**^△△^**^Ct^ method [21]. The mean miRNA level from three real-time quantitative PCR experiments was calculated for each case.

### Western blot analysis

Cells were harvested at indicated time points and lysed in RIPA buffer (Sigma). Western blot analysis was performed with the use of conventional protocols as described previously [22]. Nuclear and cytoplasmic proteins were extracted in accordance with the manufacturer's instructions (Pierce Biotechnology). The antibodies and dilutions used included anti-β-actin (AC-15; 1:2000; Sigma), anti-Keap1 (D1G10; 1:1000; Cell Signaling Technology), anti-Nrf2 (D1Z9C; 1:1000; Cell Signaling Technology), anti-LaminB (M-20; 1:1000; Santa Cruz), anti-GFP (A00185.01; 1:1000; Santa Cruz). After extensively washed, the membranes were incubated with anti-mouse or anti-rabbit IgG-horseradish peroxidase conjugate antibody (Zhongshan Company) for 1 h at room temperature and developed with a Luminol chemiluminescence detection kit (Santa Cruz). Membranes were reprobed for β-actin antibodies for normalization and accurate quantification. Protein expression level was quantified by using a Gel EDAS 293 analysis system (Cold Spring USA Corporation) and Gel-Pro Analyzer 3.1 software (Media Cybernetics).

### Reporter assay

To measure the transcriptional activity of Nrf2, reporter assays were performed using the ARE promoter reporter construct. Cells were transfected in 24-well plates with pGL3-ARE and/or pcDNA3-HA-Nrf2. The total amount of transfected DNA was kept constant by adding pCL3-basic and pcDNA3-HA plasmids. Transfection efficiencies were estimated using co-transfected pEGFP-C1 (20 ng). The protein level of GFP was detected by western blotting. After transfection, cells were treated with or without MSA for 48 h. Then, Firefly luciferase activity was determined using the Dual-luciferase reporter assay system (Promega). All results were expressed as means ± S.D. for independent triplicate cultures.

To demonstrate the *miR-200a* directly regulates Keap1 expression by binding to its 3′-UTR, reporter assays were performed using Keap1 3′-UTR-wt and Keap1 3′-UTR-mut. Cells in 24-well plates were transfected with the indicated plasmids (300 ng) and the internal control plasmid pRL-SV40 (2 ng). After transfection, cells were treated with or without MSA for 24 h. Then, luciferase activity was determined as mentioned above. All results were expressed as means ± S.D. for independent triplicate cultures.

### ChIP

Using Chromatin immunoprecipitation Kit & Shearing Kit (Active Motif), ten millions of KYSE150 cells were cross-linked and lysed and chromatin was sheared to 200–700 bp fragments. Sheared chromatin–DNA mixture was incubated with 4 μg of KLF4 (H-180, Santa Cruz), negative control IgG antibody per reaction overnight at 4°C. Cross-links were reversed and protein was removed by digestion with proteinase K. Purified DNA was used for RT-PCR. Using the following primers: *miR-200a* promoter (244-bp,–1048—-804) forward: 5′-GCTCACCCTTGCAGGTCTCC-3′ and reverse: 5′CCCGAAACCCAGCCGCATC-3′.

### Statistical analysis

SPSS for Windows (SPSS Inc.) was used for statistical analysis. A Student's two-tailed non-paired *t*test was used to determine significant differences between treatment and control values in all the experiments. Values of *P*<0.05 were considered statistically significant.

## RESULTS

### MSA could activate Keap1/Nrf2 pathway in ESCC cells

We firstly examined the impact of MSA on endogenous Keap1 expression in ESCC cell lines. In both KYSE180 and KYSE150, MSA treatment significantly down-regulated Keap1 and up-regulated Nrf2 at protein level compared with the control cells without MSA treatment respectively (Figure 1A). We further examined Nrf2 and Keap1 expression in cytoplasm and nuclear extracts with western blotting. Upon MSA treatment, we found an elevated expression level of Nrf2 located in the nucleus and a decrease expression level of Keap1 both in the cytoplasm and in the nucleus, in comparison with the nuclear protein loading control (lamin B) and the cytoplasm loading control (β-actin; Figure 1B). Meanwhile, the luciferase reporter ARE had been used to characterize Nrf2 transcriptional activity in KYSE150 cells with or without MSA treatment. As shown in Figure 1(C), co-transfection with Nrf2 could enhance the ARE promoter activity much higher with MSA treatment than without.

**Figure 1:**
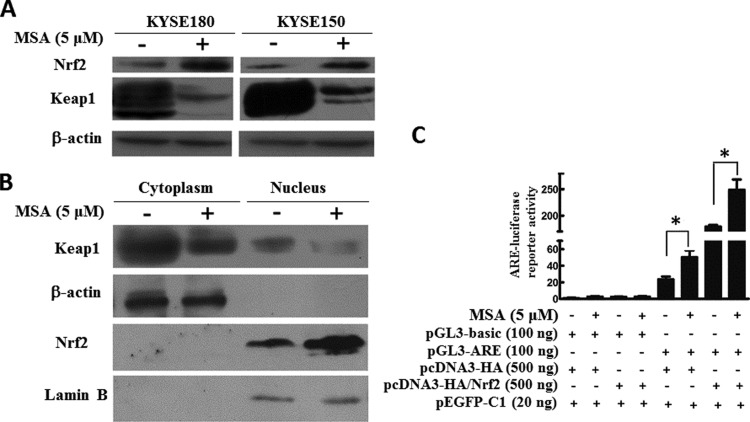
MSA activated Keap1/Nrf2 pathway in ESCC cells (**A**) KYSE180 and KYSE150 were treated with MSA (5 μM) for 24 h. Then, total cell lysates were prepared. Western blotting was performed to examine Keap1 and Nrf2. β-Actin was used as a loading control. (**B**) KYSE150 cells were treated with MSA (5 μM) for 24 h. Then, protein lysates were prepared. Keap1 and Nrf2 were detected in the cytoplasmic and nuclear extracts by western blot. β-Actin was used as a loading control of cytoplasmic protein and lamin B was shown as a loading control of nuclear protein. (**C**) KYSE150 cells were transfected with 100 ng of pGL3-ARE or pGL3-basic and 500 ng of pcDNA3-HA or pcDNA3-HA/Nrf2 plasmids with or without MSA treatment respectively. The pEGFP-C1 (20 ng) plasmid was co-transfected to normalize transfection efficiency. The luciferase activities were measured 48 h after transfection. Means ± S.D., *n*=3, *P<0.05.

In all, these findings demonstrated that up-regulation of Nrf2 in the nucleus caused by MSA plays its transcriptional role in ESCC cells.

### MSA activates Keap1/Nrf2 pathway via up-regulating *miR-200a*

Previous study has showed that Keap1 is a direct target of *miR-200a* [16,18]. In order to test if MSA inhibited Keap1 through *miR-200a* at the post-transcriptional level, KYSE 150, KYSE 410, KYSE 180 and KYSE 510 cells were treated with MSA (5 μM) for 24 h, the level of *miR-200a* was detected by real-time PCR afterwards. As shown in Figure 2(A), MSA could significantly induce *miR-200a* expression in ESCC cells.

**Figure 2:**
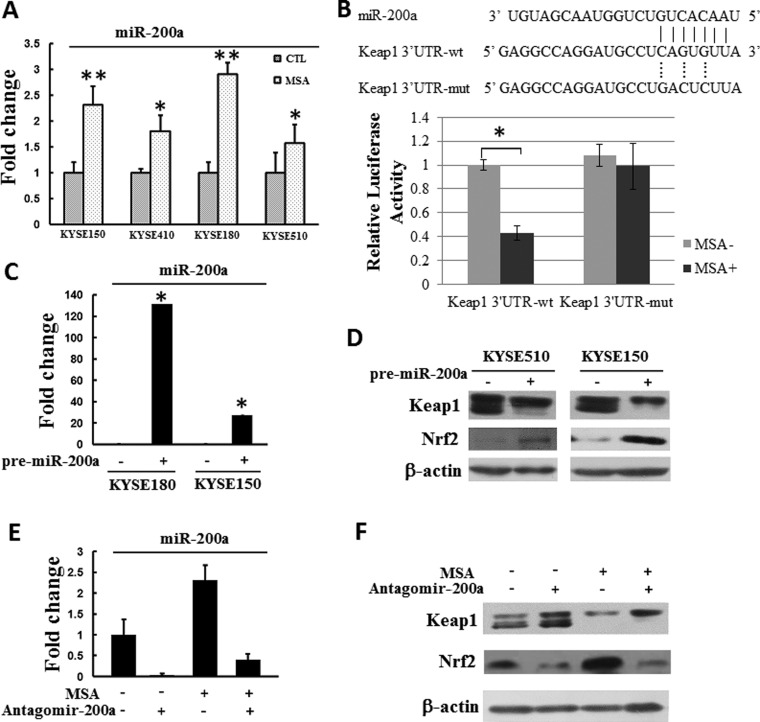
MSA activates Keap1/Nrf2 pathway via up-regulating *miR-200a* (**A**). KYSE150, KYSE 410, KYSE 180 and KYSE510 were treated with or without MSA (5 μM) for 24 h, then the expression level of *miR-200a* was detected by real-time PCR. U6 was used as an internal control. The level of *miR-200a* in each cell lines without MSA treatment was designated as unit 1 respectively. Means ± S.D., *n*=3, *P<0.05, **P<0.01. (**B**) Predicted duplex formation between human Keap1 3′-UTR and *miR-200a*. Luciferase activity of Keap1 3′-UTR wild-type (Keap1 3′-UTR-wt) or mutant (Keap1 3′-UTR-mut) reporter gene in KYSE150 cells with or without MSA treatment (5 μM, 24 h) were detected. Mean ± S.D. (*n*=3), *P<0.05. (**C**) KYSE180 and KYSE150 cells were transfected with either 30 nM Pre-miR *miR-200a* precursor or a Pre-miR negative control miRNA precursor. miRNA was extracted 24 h after transfection. Real-time PCR was performed to examine *miR-200a* level. The level of *miR-200a* in the corresponding cells that transfected with the negative control was designated as unit 1 respectively. Means ± S.D., *n*=3. (**D**) KYSE510 and KYSE150 cells were transfected with *miR-200a* precursor (30 nM) or pre-miR negative control (30 nM) respectively. Keap1 and Nrf2 protein levels were detected 24 h after transfection. β-Actin was used as a loading control. (**E**) *miR-200a* level in KYSE150 cells after the administration of MSA (5 μM) and/or antagomir-200a (20 μM) was measured by real-time PCR. U6 was used as the endogenous control. The level of *miR-200a* without MSA and antagomir-200a treatment was designated as unit 1. Means ± S.D., *n*=3. (**F**) The levels of Keap1 and Nrf2 in KYSE150 cells treated with MSA (5 μM) following antagomir-200a (20 μM) transfection were detected by western blot. β-Actin was used as a loading control.

In order to test whether *miR-200a* could directly regulate Keap1 by binding to its 3′-UTR in ESCC cells, the Keap1 3′-UTR was cloned into the pMiR-Report vector, downstream the luciferase gene (pMiR-Report/Keap1 3′-UTR-wt, abbreviated to Keap1 3′-UTR-wt). For control group, a mutated Keap1 3′-UTR with three bases mutations from the site of perfect complementarity was cloned (pMiR-Report/Keap1 3′-UTR-mut, abbreviated to Keap1 3′-UTR-mut). As shown in Figure 2(B), transfection of Keap1 3′-UTR-wt following with MSA treatment led to a decrease in the luciferase activity in KYSE 150 cells. In contrast, MSA treatment did not influence the luciferase activity of Keap1 3′-UTR-mut, demonstrating that mutation of the *miR-200a*-binding site in the Keap1 3′-UTR abolished the inhibitory effect of *miR-200a* to regulate its expression. In addition, transfection of Pre-miR *miR-200a* precursor could effectively express *miR-200a* (Figure 2C) and increased expression of *miR-200a* led to a reduced production of Keap1 at protein level (Figure 2D). Furthermore, we also treated KYSE150 cells with antagomir-200a and re-examined the impact of MSA treatment. The results showed that antagomir-200a could attenuate MSA treatment-induced Keap1 down-regulation and Nrf2 up-regulation (Figures 2E and 2F).

In summary, these results showed that Keap1 was a direct target of *miR-200a* in ESCC cells. Also, MSA could up-regulate the expression of *miR-200a*.

### MSA-induced *miR-200a* expression depended on the mediation of KLF4

Our previous study reported that KLF4 could be up-regulated by MSA in ESCC cells [19]. We examined the genomic sequences in the 5′-direction of the *mir-200a* gene. Intriguingly, a putative binding site at the ∼1 kb upstream of the *mir-200a* was observed for KLF4 transcription factor. To determine whether KLF4 could up-regulate *miR-200a* in human ESCC cells, quantitative real-time PCR was performed. As shown in Figures 3(A) and 3(B), MSA could up-regulate the mRNA level of KLF4. When KLF4 expression was inhibited by using KLF4 siRNA, MSA could not induce *miR-200a* expression anymore. The results of western blotting showed that knockdown of KLF4 could abolish MSA treatment-induced Keap1 down-regulation and Nrf2 up-regulation. Furthermore, ChIP assay showed that MSA could significantly increase the binding of KLF4 to the *miR-200a* promoter region in ESCC cells. All of these results indicated that MSA-induced *miR-200a* expression was dependent on the mediation of KLF4.

**Figure 3:**
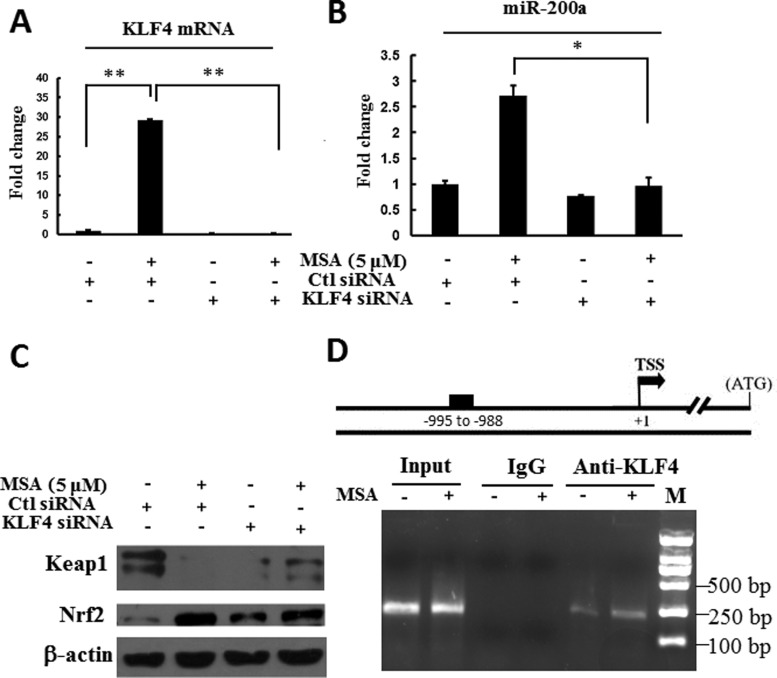
KLF4 mediated *miR-200a* up-regulation induced by MSA in ESCC cells KYSE180 cells were transfected with KLF4 siRNA or control siRNA for 48 h. Then, the cells were treated with or without MSA (5 μM) for additional 24 h. Total RNA was extracted. KLF4 mRNA (**A**) and *miR-200a* level (**B**) were determined by real-time PCR. The level of KLF4 or *miR-200a* in KYSE180 cells with control siRNA transfection and without MSA treatment was designated as unit 1 respectively. Means ± S.D., *n*=3. (**C**). The levels of Keap1 and Nrf2 in KYSE180 cells treated as indicated were detected by western blot. β-Actin was used as a loading control. (**D**). ChIP assay result showed the KLF4 that associated with *miR-200a* prompter. KYSE150 cells were treated with MSA (5 μM, 24 h). Then, the lysates were immunoprecipitated by KLF4 antibody and–1048—-804 region of *miR-200a* promoter was amplified with specific primers.

## DISCUSSION

ESCC occurs at very high rates in certain regions of China [23]. There are increasing evidences demonstrating that selenium could act as a potential anti-oesophageal cancer agent, but the precise mechanisms involved are still not completely understood. Significant risk factors for ESCC include tobacco smoking and alcohol consumption [24]. It has been reported that carcinogenesis induced by tobacco and alcohol is mediated, at least in part, by oxidative stress [25]. Oxidative stress is a term used to denote the imbalance between the concentrations of reactive oxygen species (ROS), reactive nitrogen species (RNS) and the defence mechanisms of the body. There is ongoing oxidative stress in the human body. Such an imbalance plays a pivotal role in many pathological conditions, including cancer [25]. The Keap1/Nrf2 signal pathway has been considered to protect cells against carcinogenesis and attenuate cancer development via neutralization ROS or carcinogen [26,27]. MSA, as a potent second-generation selenium compound, is a known antioxidant and chemopreventive agent. Our previous study demonstrated that MSA could inhibit HDAC activity in ESCC cells [19]. In the present study, for the first time, our results indicated that MSA strongly inhibited Keap1 expression via *miR-200a* and induced Nrf2 protein expression and nucleus accumulation in ESCC cells. In agreement, the regulation of Keap1 expression by *miR-200a* has been previously studied in breast cancer [16]. Besides, there are evidences that HDAC inhibition could activate Nrf2 activation by inhibiting Keap1 expression and the decrease in Keap1 expression was an indirect effect [28]. Thus the miRNA inhibition might be an underlying mechanism to explain the phenotype. Furthermore, a recent study showed that Nrf2-knockdown mice were more susceptible to 4-nitroquinoline-1-oxide (4NQO)-induced tongue and oesophageal carcinogenesis than wild-type mice, suggesting that Keap1/Nrf2 system is an important defence mechanism for upper aerodigestive tract carcinogenesis [12]. These data support the idea that the chemopreventive property of MSA in oesophageal carcinogenesis may depend on the activation of Keap1/Nrf2 pathway. *In vivo* studies are needed to further assess the chemopreventive effect of MSA.

It was reported that KLF4 could be up-regulated by MSA in human prostate cancer and ESCC cells [19,29]. In our study, KLF4 regulation of *miR-200a* expression was examined in ESCC cells. Previous study has validated that HDAC4 could inhibit the expression of *miR-200a* and reduce the histone H3 acetylation level at the *mir-200a* promoter through a Sp1-dependent pathway in hepatocellular carcinoma [30]. Our earlier experimental evidences support the idea that MSA could enhance the acetylation of histone H3 in ESCC cells [19]. An interesting possibility is that MSA-induced histone H3 acetylation may directly bind to the *mir-200a* promoter region and thus increased *miR-200a* expression in ESCC cells. Therefore, the possible involvement of histone H3 acetylation in the regulation of *miR-200a* deserves further investigation.

In conclusion, our study not only demonstrated that MSA could activate Keap1/Nrf2 pathway via up-regulating *miR-200a* but also found a novel mechanism by which *miR-200a* expression was regulated in ESCC cells.

## Abbreviations

ARE: antioxidant response element
ESCC: oesophageal squamous cell carcinoma
HDAC: histone deacetylase
Keap1: kelch-like ECH-associated protein 1
KLF4: Krüpple-like factor 4
MSA: methylseleninic acid
mut: mutation
Nrf2: nuclear factor E2-related factor 2
RNS: reactive nitrogen species
ROS: reactive oxygen species
SAHA: suberanilohydroxamic acid
Sp1: specificity protein 1
wt: wild type
